# Trusted Cameras on Mobile Devices Based on SRAM Physically Unclonable Functions †

**DOI:** 10.3390/s18103352

**Published:** 2018-10-08

**Authors:** Rosario Arjona, Miguel A. Prada-Delgado, Javier Arcenegui, Iluminada Baturone

**Affiliations:** Instituto de Microelectrónica de Sevilla (IMSE-CNM), Consejo Superior de Investigaciones Científicas (CSIC), Universidad de Sevilla, Américo Vespucio, 28, 41092, Seville, Spain; prada@imse-cnm.csic.es (M.A.P.-D.); arcenegui@imse-cnm.csic.es (J.A.); lumi@imse-cnm.csic.es (I.B.)

**Keywords:** cameras on mobile devices, camera security, camera identification, trusted cameras, physically unclonable functions (PUFs), SRAM PUFs

## Abstract

Nowadays, there is an increasing number of cameras placed on mobile devices connected to the Internet. Since these cameras acquire and process sensitive and vulnerable data in applications such as surveillance or monitoring, security is essential to avoid cyberattacks. However, cameras on mobile devices have constraints in size, computation and power consumption, so that lightweight security techniques should be considered. Camera identification techniques guarantee the origin of the data. Among the camera identification techniques, Physically Unclonable Functions (PUFs) allow generating unique, distinctive and unpredictable identifiers from the hardware of a device. PUFs are also very suitable to obfuscate secret keys (by binding them to the hardware of the device) and generate random sequences (employed as nonces). In this work, we propose a trusted camera based on PUFs and standard cryptographic algorithms. In addition, a protocol is proposed to protect the communication with the trusted camera, which satisfies authentication, confidentiality, integrity and freshness in the data communication. This is particularly interesting to carry out camera control actions and firmware updates. PUFs from Static Random Access Memories (SRAMs) are selected because cameras typically include SRAMs in its hardware. Therefore, additional hardware is not required and security techniques can be implemented at low cost. Experimental results are shown to prove how the proposed solution can be implemented with the SRAM of commercial Bluetooth Low Energy (BLE) chips included in the communication module of the camera. A proof of concept shows that the proposed solution can be implemented in low-cost cameras.

## 1. Introduction

Cameras are employed in many applications that require high security, like the widely used surveillance systems. Nowadays, many networked cameras are not part of closed circuit television (CCTV) systems or closed image sensor networks. Instead, they are connected to larger and public networks such as the Internet. This implies an increase of the security risks because cameras are attractive targets for cyberattacks [1]. In addition, many cameras are not static, but they are placed on mobile devices since current daily life is featured by mobility. The problem is that cameras on mobile devices have constraints in size, computational capabilities, and power consumption, so that their security has to be achieved at low cost [2].

Inclusion of cameras in mobile devices has spread their application domains. In military applications, for example, tiny cameras are included on the helmets of troops to help soldiers in the battlefield. Cameras can identify the objects in front of the soldiers to detect threats and to share information among them and the officers in charge of the mission. In novel surveillance applications, spy drones with cameras are being increasingly used by police units, government sectors or officials and military forces. Cameras in civil action applications related to sports such as cycling, surfing, running, etc., are highly demanded. The Internet of Things cameras, such as baby monitors or telecare cameras, and the cameras used in medical devices, such as endoscope cameras, are also being increasingly used [3,4]. 

Since in most of the above-mentioned applications the receivers do not see the scenes that the camera is capturing, they have to trust in the camera. A requirement of any trusted communication is the authentication of the sending point, that is, the receiver should be able to verify that the received images have been captured by a previously identified camera. This is the first link of a digital chain of custody. Camera identification is an important branch of multimedia forensics that aims to associate specific captures to the source camera from which they were taken. As a matter of fact, digital content can be used as evidence in judicial proceedings provided that the camera is proven to have been capturing the criminal activity. Camera identification is also interesting to avoid counterfeiting cameras. Counterfeiting is a big problem, not only for original camera manufacturers, who can lose lot of money, but also for consumers, because the lower quality of fake cameras increases the probability of application failures.

In many cases, the information acquired by the camera is sensitive. This happens if they are used by police or military forces, or if they can threat personal privacy and anonymity (since it is possible, for example, to track and identify people with digital cameras). Attackers may want to intercept the information transmitted by the camera in order to gain information. In these cases, not only camera identification is required for trusted communication but also information encryption is required to avoid sniffing attacks. Confidentiality of the images is also imposed by legal regulations in order to preserve sensitive personal data [1].

In addition, if information sent by a camera is not authenticated, man-in-the-middle attacks can be carried out. The images can be altered and false images can be injected to show a situation that replaces the real scene. An example of man-in-the-middle attack is the facial reenactment described in [5], where the content of the video captured by a camera is modified in real time before being received (the facial expressions of a target actor are changed by the expressions of a source actor). There are also popular software tools, like Virtual Webcam, ManyCam or Magic Camera, which are able to modify the images captured and to simulate real-time captures. To avoid man-in-the-middle attacks, information integrity is required. 

Another well-known cyberattack is to take the remote control of the camera and to record data without the knowledge and permission of the camera’s owners. For example, the control of a camera connected to the Internet is usually taken by obtaining the password employed for the WiFi connection. These attacks are produced even if the cameras are in the stand-by (or energy-saving) mode because they are designed to maintain a wireless connection in any circumstance, for example by using the Bluetooth communication module. A well-known Distributed Denial-of-Service (DDoS) attack was performed in October 2016 by means of installing the Mirai malware on a large number of devices [6]. In this case, remotely controlled bots, mostly Internet-connected cameras, were employed to spread the malware. In order to avoid this, the firmware (the code) to be executed by the camera should be proven to be authentic [7,8,9].

Many techniques have been proposed for camera identification in the last two decades [10,11,12,13,14,15,16,17,18,19,20,21,22,23,24,25,26,27]. An identifier is extracted and associated with a camera as a fingerprint that makes it distinguishable from another one (of the same brand or even of the same model). Camera identifiers are features that can be extracted from three types of sources: (1) metadata assigned to the camera; (2) captures from which to obtain characteristics defined by the electronics components and processing algorithms of the camera; and (3) electronic components from which to extract intrinsic characteristics of the camera hardware. In the latter case, Physically Unclonable Functions (PUFs) have been reported to generate unique, distinctive and unpredictable identifiers produced by the manufacturing process variability of the hardware [28]. In contrast to metadata (which are externally assigned to the camera), or identifiers extracted from the captures (which depend on the type of external scenes), hardware-based PUFs are intrinsic to the camera and are directly extracted from the camera hardware [23,24,25,26,27]. These PUFs allow adding security functionalities at a low cost, without burdening the processing unit of the camera. This is why this paper presents a camera identification technique using hardware-based PUFs.

Encryption algorithms should be selected carefully to provide real-time performance with low-cost cameras on mobile devices, which have constrained computing and memory resources. Digital Rights Management (DRM) schemes are widely used to protect video streams on the Internet, but they employ cryptographic techniques that are computationally intensive for low-cost cameras. Cryptographic techniques (symmetric and asymmetric) are the standard techniques to provide security. The symmetric cryptography techniques, which employ the same key to cipher and decipher, have the problem of secret key communication through non-secure channels, and the vulnerability to repudiation attacks, because they do not use digital signatures. On the other side, the asymmetric techniques, which employ a pair of public–private keys for each communication extreme, are computationally more complex than symmetric techniques, particularly for multimedia data, and require larger key sizes (generally, the double). Hence, asymmetric techniques are usually employed to interchange keys through non-secure communication channels and to create digital signatures (which provide non-repudiation), and symmetric techniques are used to cipher and decipher the data [1,29,30]. Several solutions based on chaos theory, cellular automata and DNA computing have been reported to authenticate encrypted images [31]. However, since they do not follow cryptographic standards, their security can be compromised [32]. This is why this paper focuses on standard and symmetric cryptographic techniques.

The contribution of this work is the proposal of a lightweight solution for trusted cameras based on binding the software executed in the camera to the hardware of the same camera (without inclusion of additional hardware). The solution is designed as a complete cryptographic solution that provides camera identification, information confidentiality and integrity, as well as a protocol that protects the communication with the camera, which is particularly interesting to carry out trustworthy camera control actions and firmware updates. PUFs are extracted from the Static Random Access Memories (SRAMs) included in the camera hardware to generate the physical identity of the camera, to obfuscate cryptographic keys, and to obtain nonces (numbers used only once). In summary, the proposed solution provides: (a) authentication by guaranteeing the origin of the data through the camera identifiers extracted from PUFs; (b) information confidentiality by ciphering the messages with secret keys that are not stored in the camera but reconstructed whenever needed with the identifiers extracted from PUFs; (c) information integrity by means of Message Authentication Codes (MACs); and (d) freshness in the communication through the nonces extracted from PUFs. Experimental results obtained from the SRAM-based PUFs found in the low-cost cameras validate the proposal. A proof of concept shows that the proposed solution can be implemented in low-cost cameras.

A preliminary version of this work appears in [27]. This work does not provide details of the operation modes of the camera and does not describe any communication protocol with the camera. A standard off-the-shelf SRAM is used as PUF, which is not included in any unit of the camera, and experimental results are provided for the PUF working at nominal operation conditions. 

The new contributions of this paper are the following. It summarizes the camera identification techniques proposed in the literature and mentions the motivations to select SRAM PUFs as camera identifiers. This is the focus of Section 2. Section 3 details the algorithms executed in enrollment and normal operation phases to generate camera identifiers, nonces and secret keys which are employed in standard cryptographic algorithms. A trustworthy communication protocol is proposed in Section 4 which satisfies authentication, confidentiality, integrity, and freshness by means of authenticated encryption and key derivation techniques based on the trusted cameras previously described. This paper employs as PUF the SRAM included in the communication unit of the camera and more exhaustive experimental results are included, particularly with the PUF working at non-nominal operation conditions. These experimental results concerning reliability, uniqueness, unpredictability and randomness of the identifiers and nonces extracted from the SRAM PUF are shown and discussed in Section 5. A prototype of a low-cost trusted camera is presented also in Section 5 as a proof of concept. Finally, conclusions are given in Section 6.

## 2. Summary of Camera Identification Techniques

Several camera identification techniques have been reported in the literature. The simplest ones employ metadata, related to information stored in the camera about the conditions of the image acquisition. The most common metadata are Exif (Exchangeable images file format), which usually include the version of the camera software, the image resolution, date and time and, in some cases, the GPS coordinates of the image acquisition. A main drawback of these techniques is to be very vulnerable to malicious attacks. The metadata file can be modified easily, for example by using multimedia processing tools. A similar approach is to assign a specific number to each camera, for example, identifying a camera by its MAC (Media Access Control) address. However, a MAC address can also be faked easily.

Other techniques extract identification features from the camera captures, taking advantage that the software and hardware components of the camera leave traces in the content of the images [10]. Identification features can be extracted from statistical information, color components, image quality metrics, and frequency domain of the images. Generally, several features are combined to obtain more accuracy in identification. A feature selection algorithm is usually employed to consider the most representative features, and a complex classification process, generally based on a Support Vector Machine (SVM), is applied. In [10], 16 statistical, 12 color, 40 quality, and 81 wavelet features are considered. The 16 statistical values (mean, median, maximum, minimum, variance, kurtosis and skewness) are computed for rows and columns, as well as their ratio (between rows and columns). The 12 color features are extracted from the pixels’ average values for each RGB channel, correlation pairs between RGB bands, neighbor distribution center of mass for each color band, and energy ratios between RGB pairs. In order to detect the 40 quality differences in brightness, sharpness and color quality, measures based on the pixels differences, correlation and spectral distance are taken. Prior to the computation of the image quality metrics, a filtering should be applied to reduce the noise of the original image (for example, a Gaussian filter to perform image smoothing). The 81 wavelet features are obtained from the pattern noise image (from 3 color channels × 3 wavelet components × 9 central components). Therefore, this approach requires high computing resources.

Deep learning is also applied to camera identification, specifically for identification of the manufacturer and the model of the camera. In [11], a fixed high-pass filter suppresses image content (such as interferences caused by image edges and textures) and extracts low-level features. The filter is applied to each color channel separately. Then, a wavelet-based denoising filter is applied and the resulting image is fed to a Convolutional Neural Network. In [12], the fixed high-pass linear filter is replaced by a nonlinear filter. The feature maps produced by a set of constrained convolutional filters concatenated with the nonlinear feature residuals are then passed to a regular convolutional layer. The approaches based on deep learning require not only higher computing resources than the previously commented approach but also big databases for training.

The lens system of a camera has been also employed to generate identifiers since they can introduce distinctive aberrations, such as astigmatism, spherical, coma, radial distortion, field curvature, chromatic aberrations, or dust particles. The drawback of these aberrations is that their permanence in time is reduced, which limits its use as camera fingerprints [13].

Other authors consider the interpolation algorithms used to obtain the Color Filter Array (CFA). The configuration of the CFA filters, the demosaicing algorithm, and the color processing techniques introduce significant differences between cameras of different manufacturers. However, this approach is not efficient at differentiating between different models from the same manufacturer [13].

Regarding identification techniques based on the image sensor, the most extended ones employ the Sensor Pattern Noise (SPN). It is the spatial pattern formed by the minor changes in the intensity of the pixels caused by the imperfections of the sensor manufacturing process, including the inhomogeneity of silicon wafers. In [14], the dark current noise component (also called Fixed Pattern Noise, FPN) is employed. The dark current noise is a signal collected from the sensor when it is not exposed to light and, thus, dark current can only be extracted from dark frames. This limits the method because camera identification is not possible from regular (non-dark) frames. In addition, some consumer cameras suppress this noise automatically by subtracting a dark frame from every image they take. The most used noise component is named Photo Response Non Uniformity (PRNU) [15], which is determined by the different sensitivity of the pixels to light. Not every pixel of the sensor is identical and will therefore respond differently to the same amount of light. The amount of photons converted in electrons by the pixels is associated with the photosensitive area and the presence of imperfections in the sensor chip. Thus, the PRNU is a multiplicative factor of the photoelectron number. In order to determine the PRNU of a camera, the first step is the application of an averaging operation to several images. In a second step, the noise is extracted from the averaged frame by applying a filtering operation. The performance of the PRNU extractor depends on the choice of that filtering. Wavelet transform, sparse 3D transform-domain collaborative filtering or context-adaptive interpolation algorithms are considered in the literature. Then, the noise pattern is obtained by subtracting the averaged frame without the noise from the averaged frame with the noise. In addition, spectrum equalization algorithms are considered to detect and suppress the peaks created by other artifacts (such as CFA patterns), which are similar in cameras of the same model or brand and thus survive the averaging operation. Once the approximated PRNU noise is obtained, the presence of the reference pattern in the image is evaluated by using correlation. 

PRNU is effective when the images have good quality and are acquired under specific conditions. Since the noise contains not only the PRNU information but also traces of scene details, flat-field images (e.g., blue sky) are preferred. However, flat-field images are not always available and images with varying scene details have to be considered. In this case, the impact of scene details should be suppressed by averaging a higher number of images [16]. In addition, the use of PRNU is not very efficient in low-quality cameras because they can introduce noise components that affect the PRNU estimation [17].

From a computational point of view, the dimensionality of PRNUs is as large as the original image. Thus, the storage space, the time to access the memory, and the matching complexity are considerable. In order to reduce PRNU dimensionality, decision trees [18], binarization [19], PCA (Principal Component Analysis) and LDA (Linear Discriminant Analysis) techniques [20], and random projections [16,21] have been applied. In the case of decision trees, the number of matchings is reduced but matching errors tend to increase when a large number of PRNUs are stored. Binarization reduces the storage in memory, but it degrades the matching accuracy due to information loss. PCA and LDA are applied to extract more compact PRNU representations. The disadvantage of this proposal is that the system needs to be re-trained if PRNUs from new cameras have to be included. Dimensionality is also reduced by means of random projections, which are not based on learning and do not require training. However, since the subspace is randomly selected, the representation is not optimal and the matching accuracy is compromised. Despite the complexity of the PRNU extraction process, the separation between intra-class (measurements from the genuine camera) and inter-class distributions (measurements from impostor cameras) is not complete [20].

The use of high frequency components of the PRNU pattern estimated from raw photos acquired in controlled conditions is proposed in [22] as a weak PUF. In the enrollment phase, PRNU information compressed with adaptive random projections is used to obfuscate a random binary sequence encoded with polar codes and to generate a secure sketch. In the authentication phase, only the genuine camera is able to recover the original binary sequence from the secure sketch.

Recent works propose the use of Physically Unclonable Functions (PUFs) to extract bit strings from the response of specific hardware components included in the camera. These bit strings can be employed as camera identifiers because PUFs allow generating unique, distinctive and unpredictable identifiers produced by the manufacturing process variability of the hardware [28].

In [23], the response of the pixels in a charge-coupled device (CCD) to a defined incident light is used as PUF. The light source is conceived to be integrated into the camera circuit in combination with a movable opaque cover to prevent undesirable incident light on the CCD. The analog electrical signals emitted by the pixels are converted into digital signals which are employed as camera identifiers. The disadvantage of this proposal is that extra hardware modules should be included in the camera.

In [24], PUFs are extracted from Dark Signal Non-Uniformity (DSNU) in CMOS image sensors. The PUF response is a binary string extracted from the pixel array. Each response bit is obtained by comparing the reset voltages of two pixels. The output bit is ‘0’ or ‘1’ depending on which reset voltage is larger. The selection of the pixel pairs is determined by a digital input challenge and a seed that initializes a Linear Feedback Shift Register (LFSR). The input challenge selects the first pixel and the second pixel is selected by encrypting (XOR-ing) the input challenge with an LFSR-based stream cipher. The pixel pair is selected for the PUF response if their reset voltage difference is greater than a predefined threshold $P_{th}$, so as to ensure that the output bit is stable. Given an input challenge and a seed, the Hamming Distance between the PUF response obtained and the PUF response stored in a server is calculated. If the distance is sufficiently small, the camera is correctly identified. This approach requires additional hardware because a switch transistor should be included in each pixel of the image sensor (the measurements cannot be obtained directly from commercial CMOS image sensors). Another disadvantage is that the proposal for authentication is based on extracting the seed of the LFSR from features of captured images that are sent to the central monitor for authentication. However, if the images suffer some modification, the seed is not well recovered and authentication fails. In addition, the threshold $P_{th}$ is technology dependent and should be determined empirically. For lower $P_{th}$ values, the number of challenge response pairs is higher, but the PUF reliability is worse. In contrast, for higher $P_{th}$ values, the PUF reliability is better but the number of challenge response pairs is lower.

The proposal in [25] is similar to [24] but oriented to DVS (Dynamic Vision Sensor) cameras. In order to isolate the PUF response and to prevent the DVS events from interfering with the PUF operation, three transistors are added to each pixel. This issue introduces more complexity than the previous proposal.

In [26], camera identifiers are based on PUFs extracted from ideally exact ring oscillators (ROs). Two ROs are selected using multiplexers and their frequencies are quantified with two counters. Both counters stop when another reference counter reaches a predefined value. The values of the two counters are compared and a response bit is generated depending on the positive or negative value of the difference. The disadvantage of RO-based PUFs is that additional hardware (the ROs) should be specifically included in the camera. In [26], ROs are implemented in a trusted visual sensor node based on a field programmable gate array (FPGA). Another drawback is the extra power consumption required by the ROs to generate the PUF response.

Most camera identification techniques summarized above are not integrated under a complete standard cryptographic solution. The camera identifier is generated to authenticate the origin of the images but there are no specific proposals that describe exhaustively how the camera could satisfy not only authentication but also confidentiality, integrity, and freshness.

In this work, we present a trusted camera based on SRAM PUFs extracted from the hardware modules of the camera, without extra hardware required (SRAMs are readily present in many parts of a camera), with very low extra power consumption, with a lightweight processing cost, and integrated into a complete cryptographic solution.

## 3. A Proposal of Trusted Cameras Based on SRAM PUFs

The proposed trusted cameras are identified by their SRAM PUFS. The extraction of PUFs from SRAMs is based on the start-up values obtained by powering up the memory. Each SRAM cell is a bistable circuit whose logic memory functionality comes from two cross-coupled inverters. A write operation forces the SRAM cell to transition towards one of the two stable states (‘0’ or ‘1’). If the cell is powered-up and no write operation is carried out, the positive feedback between the two inverters leads the cell to the start-up value imposed by the inverter which begins to conduct. Ideally, the two inverters are identical, but the random variations in the manufacturing process make them different so that one of them is the first to conduct in each cell. Manufacturing process variations are unique of each SRAM, unpredictable, and difficult to be cloned physically or modeled mathematically. Hence, the bit strings composed of SRAM start-up values meet the requirements of PUF responses [28,33,34,35].

**Figure 1:**
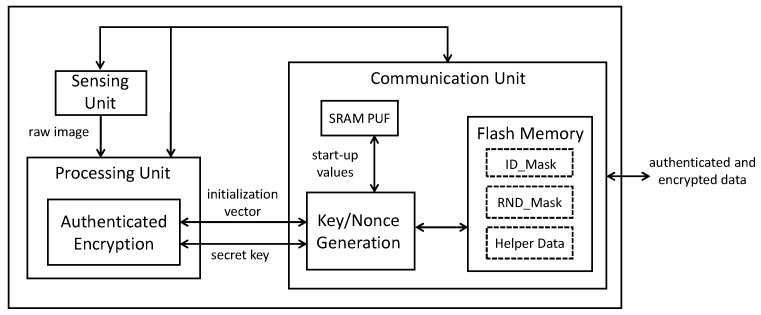
Block diagram of a trusted camera based on SRAM PUFs.

SRAM PUFs can be found in the hardware components of a camera so that no extra hardware is required, which is an advantage compared to the use of the other PUFs commented on in the previous section. The components of a camera (as illustrated in Figure 1) are the following: (1) the sensing unit where the image sensor performs the captures and converts the optical image to an electronic signal; (2) the processing unit (with the main processor), which applies different types of operations (compression or adjustments of brightness, sharpness or contrast, etc.); and (3) the communication unit, which includes input and output ports to receive or send data by employing a communication protocol (particularly Bluetooth and WiFi in the case of cameras on mobile devices). However, typically, there are SRAMs in the three units. Our proposal is to use the SRAM in the communication unit as SRAM PUF. Current cameras on mobile devices use wireless communications based on a combination of WiFi and Bluetooth Low Energy (BLE). WiFi is activated to establish communications at a high speed. However, it is much more costly in terms of power consumption. Hence, when the camera is not transmitting data, the communication module activated is the BLE in order to maintain the camera connected in a low power consumption mode. When the BLE is activated, the start-up values of its SRAM are collected as PUF responses.

The proposed trusted cameras have two main operation modes: registration or enrollment and normal operation mode. The registration or enrollment is required by any camera identification techniques in order to register the camera by its identifiers or other parameters related to them. Our proposal is to employ standard cryptographic techniques in the normal operation mode and identify the camera by its SRAM PUF, so that only a genuine camera with its SRAM PUF and its non-sensitive information stored in its non-volatile memory is able to reconstruct the cryptographic key. The non-sensitive information is related to the physical identity of the camera as will be described in the following.

### 3.1. Enrollment Phase

The use of cryptographic techniques with a lightweight processing cost requires the generation not only of good identifiers but also of nonces. Hence, the proposed trusted cameras are registered with the following steps. The first step is to select the SRAM cells that provide generally the same start-up value, which will be named herein as STB cells. For that purpose, the simple processing described in Algorithm 1 and proposed in [33] is carried out. Most of the SRAM cells are STB cells. They are considered in the STB_mask. However, the inverters of some cells are very similar so that their start-up values change due to noise, thus providing flipping bits. These cells change its values in each start-up and they are not selected for identifiers. Particularly, those which usually change their start-up values, for example half of the time, are selected to generate the nonces. They are named herein as RND cells. In Algorithm 1, the number of start-up value changes for each cell is accumulated in the STB_counter. If the STB_counter is M/2 for a cell, the cell is considered in the RND_mask, and when it is zero, it is considered in the STB_mask.

**Algorithm 1** Pseudo-code of STB and RND masks extraction**Require**: Number of measurements M  for i = 1 to M do    power down and up the SRAM    if i = 1 then      save the start-up values    else      for all the cells of SRAM do        compare the start-up value of the cell with the stored one        if cell value does not change then          do not increase the count for the cell in STB_counter        else          increase the count for the cell in STB_counter        end if      end for    end if  end for  for all the cells of SRAM do    if its count in STB_counter is 0 consider the cell in the STB_mask    if its count in STB_counter is M/2 consider the cell in the RND_mask  end for  reset all STB_counters to zero **return** STB_mask, RND_mask

The second step of the enrollment phase is to select the STB cells that provide a debiased PUF response, which will be named herein as ID cells. This step is needed because a requirement to ensure the unpredictability property of a PUF is that the number of zeros and ones in the PUF response should be the same. For that purpose, the simple processing described in Algorithm 2 is carried out. It applies the pair-output von Neumann (2O-VN) debiasing technique proposed in [36].

**Algorithm 2** Pseudo-code of 2O-VN debiasing**Require**: STB_mask  power down and up the SRAM  apply the STB_mask and obtain response from N STB cells  for j = 1 to N/2 do    if start-up-value-of-cell(2j) ≠ start-up-value-of-cell(2j-1) consider both cells in ID_mask  end for **return** ID_mask

The third step of the enrollment phase is to generate the Helper Data needed to apply a Code Offset-based Helper Data algorithm as described in [37]. Code Offset-based Helper Data algorithms employ Error Correcting Codes (ECC) to generate non-sensitive information, known as Helper Data, from sensitive data such as a cryptographic key and noisy data such as the PUF response. The complexity of the ECC is higher as the noise (flipping bits) in the PUF response is higher [33]. Since ID cells do not provide usually flipping bits (they are STB cells), a simple repetition ECC is employed, as can be seen in Algorithm 3.

The last step of the enrollment phase is to store the non-sensitive information generated (the ID_mask, RND_mask, and Helper Data) in the non-volatile memory of the camera. The sensitive information (the start-up values of the SRAM cells and the cryptographic key) are not stored anywhere. Hence, this solution is more robust to attacks than approaches that directly store the cryptographic key or the camera identifiers (for example the metadata).

**Algorithm 3** Pseudo-code of Helper Data generation**Require:** Cryptographic Key $K=\left[k_{1},\;\ldots ,k_{a}\right]$, ID_mask  power down and up the SRAM  apply the ID_mask and obtain response from ID cells  each bit of the key is repeated r times: $K_{coded}=\left[k_{11},\;\ldots ,k_{1r},\ldots ,k_{a1},\;\ldots ,k_{ar}\right]$  ID = concatenation of the start-up values of a · r ID cells  Helper Data = ID $⨁K_{coded}$ **return** Helper Data

There are hardware modules that include protection, known as Trusted Platform Modules (TPMs), which store and process sensitive data in a software protected domain (ARM TrustZone [38], Texas Instruments M-Shield^TM^ [39], or Intel® Software Guard Extensions [40]). However, they are more power hungry and expensive than the standard non-volatile memories used in our proposal. In addition, several techniques have been reported to attack and extract data from them [41] and malware has been demonstrated to be run in these hardware enclaves [42].

### 3.2. Normal Operation Phase

In addition to authentication and freshness (given by the camera identifiers and nonces extracted from SRAM PUFs), the proposed trusted cameras employ standard cryptographic algorithms to provide confidentiality and integrity.

Confidentiality is achieved by symmetric ciphering. According to how the data are ciphered, ciphers can be classified into block and stream ciphers. Block ciphers process blocks of bits while stream ciphers encrypt bits individually. Among block ciphers, AES (Advanced Encryption Standard) was approved in 2001 as a US federal standard (FIPS PUB 197) and then it was included in the ISO/IEC 18033-3 standard. Hence, AES is the dominant symmetric-key algorithm in many commercial applications. In particular, many cameras on mobile devices include a hardware module that implements AES.

Integrity of the messages is achieved by Message Authentication Codes (MACs). MACs are usually employed to obtain authentication tags (or cryptographic checksums). Block ciphers or hash functions can be employed to obtain MACs. In practice, the most popular approach is to use a block cipher such as AES in Cipher Block Chaining (CBC) mode, according to NIST 800-38A [43]. In this mode, the first iteration of the MAC algorithm is computed with the secret key, an Initialization Vector (IV) and the first block of the data to encrypt. The subsequent plaintext blocks are XOR-ed with the previous ciphertext block before they are encrypted. The MAC of the message in CBC-MAC is the output of the last round. Chaining mode is preferred to encrypt long messages such as images because each ciphertext block produced not only depends on the plaintext block (and the secret key) but also on the preceding blocks. If each block is encrypted independently instead of using chaining mode, encrypting the same plain text using the same key produces the same cipher text.

The proposed trusted cameras employ AES-CBC as standard authenticated encryption algorithm. CBC mode requires a 128-bit IV and a key with a default size of 128 bits. The IV should never be reused under the same key because it leaks some information about the first block of the plain text. In addition, the IV must be also unpredictable at encryption time. If an attacker knows the IV (or the previous block of the cipher text) before the next plain text is specified, the attacker can try to obtain the plain text of some block that was encrypted with the same key before, which is known as the TLS (Transport Layer Security) CBC IV attack.

The novelty of our proposal is that the start-up values of the SRAM included in the camera are employed to reconstruct the secret key of 128 bits and to generate the 128 bits for the IV. Whenever the key is required, the key reconstruction step is carried out as described in Algorithm 4. At key reconstruction, new start-up values are obtained from the ID cells used in the enrollment phase (known by the information in the ID_mask). The start-up values will not be exactly the same as the response used at the Helper Data generation but very similar because a low bit flipping may happen. Hence, using them and the stored Helper Data, a noisy version of the encoded secret key is obtained. Then, the decoder of the ECC is able to recover the cryptographic key. In normal operation mode, the required nonces are obtained from the start-up values of the RND cells, using the information in the RND_mask.

**Algorithm 4** Pseudo-code of key reconstruction**Require:** Helper Data, ID_mask  power down and up the SRAM  $\bar{ID}$ = new concatenation of the start-up values of the a · r ID cells  $\bar{K_{coded}}$ = Helper Data ⨁
$\bar{ID}$  *K* = ECC($\bar{K_{coded}}$) **return**
*K*

## 4. A Proposal of Trustworthy Communication Protocol with the Trusted Cameras

Let us consider a typical scenario in which a base station communicates with the camera in the mobile device in order to request the acquisition of images, to update its firmware, etc. Let us assume an adversarial model in which the attacker has full control of the communication link, that is, can modify messages, inject message forgeries, replay previously sent messages, or interrupt the communication. In addition, let us assume that the attacker has the capability to reach the camera physically and obtain the information stored in it. Not only the base station should prove that the camera is trusted but also the camera should prove that the base station and its requests are trusted so as to avoid cyberattacks aimed at malicious uses of the camera. 

The trustworthy protocol proposed herein takes advantage of the camera operation modes to implement a secure communication between the camera and the base station considering the adversarial capabilities assumed above. It follows the model of secure channels introduced in [44], in which communications over insecure links are protected through sessions with two stages. First, the camera and the base station establish an authenticated and shared secret session key. Second, the session key is used with symmetric-key cryptographic functions to protect the integrity and confidentiality of the transmitted information.

As in many other protocols, there is an initial phase that is run prior to the initiation of any communication and that produces the required initial information without any adversarial attacks. The camera executes a first enrollment phase during the manufacturing process and the first symmetric key is established between the camera and the base station, assuming no attacks. The first key established for the camera C is denoted as $K_{0}^{C}$. The camera executes Algorithm 3 and stores the Helper Data, denoted as $HD_{K0}^{C}$, in the non-volatile memory. Once the first enrollment is finished, the camera can be employed in its application context. The base station identifies the camera as the one which employs the key $K_{0}^{C}$.

**Figure 2:**
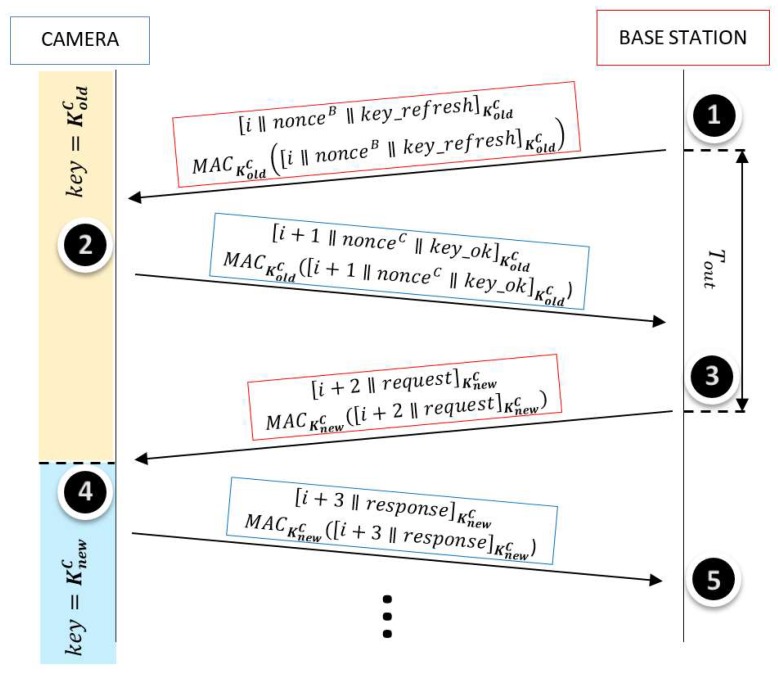
Proposed lightweight protocol.

Figure 2 illustrates the proposed protocol between a camera C and the base station. The notation used in Figure 2 to describe the operations and the message exchanges is the following:

- [a∥b] represents the plaintext “a” concatenated with the plaintext “b”.
- ${\left[c\right]}_{K}$ represents the plaintext “c” encrypted by AES-CBC using the key “K”.
- $MAC_{K}\left(d\right)$ represents the authentication tag of the message “d” using CBC-MAC with the key “K”.
- “i” represents a randomly initialized index which increases by 1 both in the sender and the receiver simultaneously after each message exchange is successfully completed.
- $nonce^{B}$ and $nonce^{C}$ represent, respectively, random numbers generated by the base station and the camera.

The symmetric keys and the IVs of the AES-CBC are refreshed based on the nonces generated for each session. Hence, in order to initiate a communication between the camera and the base station, they authenticate each other by their knowledge of $K_{old}^{C}$ and then derive a new session key $K_{new}^{C}$ and a new initialization vector $IV_{new}^{C}$. In Figure 2, the key agreement is carried out in Steps 1 and 2. A Key Derivation Function (KDF) that follows NIST recommendations is employed (in our case, it is based on AES) [45]. This function uses the previous key $K_{old}^{C}$, a nonce sent by the base station, $nonce^{B}$, and a nonce sent by the camera, $nonce^{C}$. In a generic session, a new key and IV are derived from the old ones as in Equations (1) and (2), respectively:

(1) $$K_{new}^{C}=AES_{K_{old}^{C}}\left(nonce^{B}\;⨁\;nonce^{C}\right),$$

(2) $$IV_{new}^{C}=AES_{IV_{old}^{C}}\left(nonce^{B}\;⨁\;nonce^{C}\right).$$

The interchanged messages are composed of an index (i) and a request (in the case of the base station) or a response (in the case of the camera). This is shown in Steps 3 and 4 in Figure 2. For example, the message in Step 3 can be the request of updating the firmware together with the firmware to update. The messages are ciphered with AES-CBC by the symmetric keys ($K_{old}^{C}$ at the key agreement and $K_{new}^{C}$ in the subsequent steps). In addition, the messages are authenticated with CBC-MAC.

AES-CBC is semantically secure or plaintext-indistinguishable, that is, given a target ciphertext and two candidate plaintexts, the attacker cannot guess the right plaintext with probability significantly better than 1/2 [44,46]. If the attacker listens all the transmitted information, nothing can be distinguished, since there are no equal plaintexts (they have different indexes) and the keys and IVs change in every session in unpredictable way. In addition, CBC-MAC resists chosen message attacks. Hence, since the proposed protocol applies the encrypt-then-authenticate method, it implements a secure channel, as demonstrated in [44] and analyzed in [46]. If the attacker has full control of the communication link, any modification of messages, injection of forgery, or replay of messages is detected by the recipient. The message index helps to avoid injection or replay attacks and to detect interruptions.

The camera, when receives a genuine message, is able to decipher it and to verify its integrity. If the message contains a request of images, the camera acquires the images. If the message contains a request of firmware update, the camera updates the firmware. Whenever the camera receives a fake message (the camera cannot decipher it) or an altered one (it does not verify authentication tag), the camera ignores it.

If the base station does not receive any response to its request after time $T_{out}$, it considers that the reply message is lost or that the camera has not received the message. In that case, the base station resends the same message until it receives the reply message that contains the index increased or until surpassing a given number of trials.

In order to reconstruct the new session key, the camera executes Algorithm 3 to generate the new Helper Data $HD_{Knew}^{C}$ from their SRAM PUF and the new key. Then, it removes $HD_{Kold}^{C}$ and stores $HD_{Knew}^{C}$ in Step 4, once it knows the base station has employed the new key. Similarly, in Step 5, the base station removes $K_{old}^{C}$ and stores $K_{new}^{C}$ for the next communication with camera *C*, once it knows the camera has already refreshed the key. The IV is public information that is stored without protection.

If the attacker reaches the camera physically and reads the information stored in the non-volatile memory, only Helper Data and Masks can be read, but no information about secret keys can be obtained. The focus herein is the security of the camera. The security of the base station is outside the scope of this paper. It is assumed that the base station has enough resources to implement a high security strategy.

Assuming physical access to the camera, the only way to extract information about the secret keys is to apply side-channel attacks such as Differential Power Analysis (DPA) attacks to the AES or MAC algorithms while they are operating. To avoid them, the hardware implementing those primitives in the camera should be resistant to those attacks.

## 5. Experimental Results of the Proof of Concept

### 5.1. Evaluation of Identifiers Extracted from SRAM PUFs Included in Cameras

As commented above, many cameras on mobile devices use wireless communications based on a combination of WiFi and Bluetooth Low Energy (BLE). WiFi instead of BLE is activated when communication at high speed is required (for transmission of images and videos). When the camera does not require WiFi, BLE is activated instead to maintain the camera connected in a low power consumption mode. In order to evaluate if cameras can be identified unequivocally by the SRAM PUFs available in their BLE chips, the CC2541 BLE chips from Texas Instruments were analyzed. Among the SRAMs included in the BLE chip, the 8-KB SRAM from the Intel 8051 microcontroller was selected as PUF. The Intel 8051 microcontroller is also able to access 256-KB of in-system-programmable flash through a memory arbitrator block. The Helper Data, ID-Mask, and RND_mask are stored in that non-volatile memory.

A firmware was developed with the IAR Embedded Workbench to characterize the SRAM PUF in the BLE chips. The CC-Debugger from Texas Instruments was employed, connected to the BLE chip through I2C and to a PC through USB. The MSP430^TM^ microcontroller from Texas Instruments was employed to power down and up the BLE chip so as to extract automatically the start-up values of the SRAM. The SRAM PUF was evaluated extensively in five BLE chips, taking 56,000 bits per SRAM (7000 bytes). The environmental conditions that affect mostly the start-up values of SRAMs are temperature and changes in ramp-up time (i.e., the time to reach power supply voltage value after power-up) [34], but it is supposed that the ramp-up time cannot be modified because the on-chip voltage regulator cannot be tampered. In order to evaluate the reliability of the PUF under variations in temperature, three temperature values (5 °C, 25 °C and 75 °C) were considered and 120 measurements were taken for each temperature. The climatic chamber ACS EOS 200TC was employed to control the ambient temperature.

The ID_ and RND_masks for each BLE chip were created with the first 20 measurements at 25 °C, executing Algorithm 1. The resting 100 measurements at all the temperatures were employed for evaluation. The target of this methodology is to evaluate how a camera, whose enrollment phase is carried out at nominal conditions (at 25 °C), works under different temperature conditions. 

**Figure 3:**
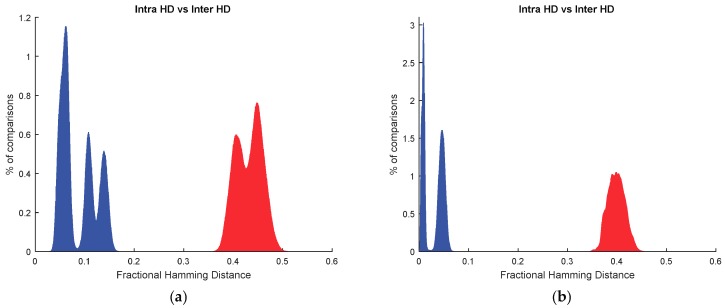
Fractional hamming distances obtained from the PUF responses (**a**) considering all SRAM cells and (**b**) considering only STB cells.

The advantages of selecting the STB cells of SRAM cells at enrollment phase are illustrated in Figure 3. In this figure, 32,768 cells were considered for each SRAM PUF, organized as 16 different responses with 2048 bits. Figure 3a shows the distribution of Hamming Distances (HD) calculated for pairs of PUF responses when all the SRAM cells are considered to generate the responses (STB as well as non-STB cells). HD computes the number of bits that are different in two PUF responses. The fractional HD, as illustrated in Figure 3, refers to the percentage of bits that are different in two PUF responses. The distribution of Hamming Distances calculated for responses from the same SRAM cells (known as genuine distribution in identification applications and intra HD distribution in the PUF literature) is shown on the left. The distribution of Hamming Distances calculated for responses from different SRAM cells (known as impostor distribution in identification applications and inter HD distribution in the PUF literature) is shown on the right. In Figure 3a, the intra HD distribution corresponds to comparisons of responses from the same SRAM cells at the three temperatures by considering all the possible combinations. The inter HD distribution is the fusion of all the possible comparisons between cells from different SRAMs.

Figure 3b shows the distribution of Hamming Distances calculated for pairs of PUF responses when only the STB cells are considered to generate the responses (filtered with the STB_mask calculated at 25 °C). The minimum percentage of STB cells found was 73.59% (which means a minimum of 41,213 STB cells for the cells evaluated in each SRAM). In Figure 3b, like in Figure 3a, 32,768 STB cells were considered for each SRAM PUF, organized as 16 different responses with 2048 bits. The intra HD distribution, at the left, contains two peaks. The distribution with the peak closer to 0 corresponds to the comparisons of responses at 25 °C. The other part of the intra HD distribution is related to comparisons of responses at different temperatures. Compared with Figure 3a, bit flipping is reduced in the genuine responses, as desired in reliable PUFs. Regarding the inter HD distribution, it is more compact than without applying classification. In any case, it can be seen that the inter HD distribution is not centered at 0.5, which means that the PUF responses do not contain the same number of ones and zeros. 

**Figure 4:**
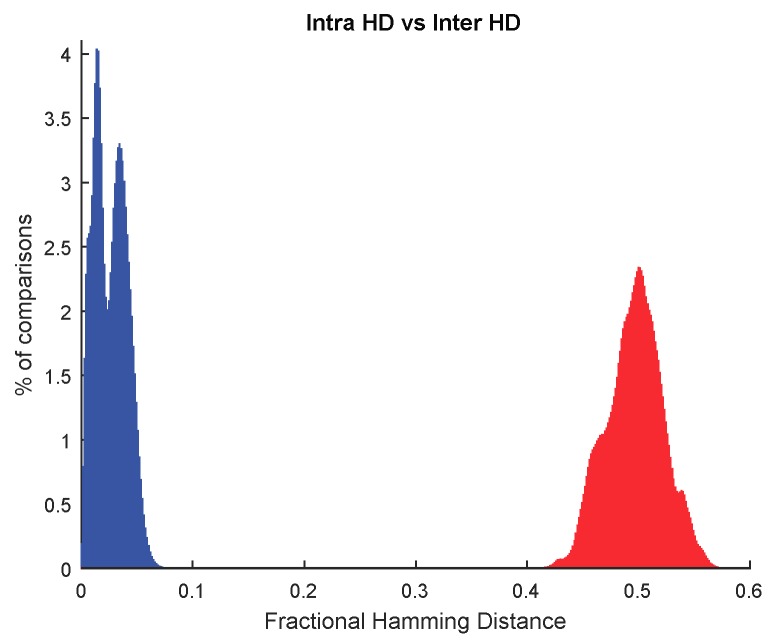
Fractional Hamming Distances obtained from the PUF responses considering only ID cells.

After applying the debiasing with Algorithm 2, the ID cells are found. 2O-VN Debiasing is very simple computationally, but it eliminates many cells. The minimum percentage of ID cells found among STB cells was 35.55% (which means a minimum of 14,651 ID cells for the cells evaluated in each SRAM). Figure 4 shows the distribution of Hamming Distances calculated for pairs of PUF responses when only the ID cells are considered to generate the responses. In this figure, 11,648 ID cells were considered for each SRAM PUF, organized as 16 different responses with 728 bits. The intra HD is shown on the left and the inter HD distribution is illustrated on the right. The intra HD distribution is similar to the result in Figure 3b since ID cells are STB cells. Thus, bit flipping is reduced and it is possible to extract reliable identifiers from the same camera. Regarding the inter HD distribution, there is a considerable improvement when using ID instead of STB cells because the distribution is centered at 0.5. Thus, identifiers from different cameras are very different. As a matter of fact, the Hamming Weight (defined as the percentage of ones in the PUF responses) has an averaged value of 49.55%, which means that distribution of ones and zeros is uniform, as required in unpredictable PUFs.

### 5.2. Obfuscation of Secret Keys by SRAM PUFs Included in Cameras

As described in Algorithm 3, Helper Data are generated from XOR-ing the PUF response with the secret encoded with a repetition ECC. Hence, Helper Data do not leak information about the secret if the PUF response is highly random. A necessary feature of random PUF responses is to be uniform, which is achieved by considering ID cells, as shown above.

Another way to evaluate the randomness of *k* PUF responses with a·r bits is to calculate the minimum entropy as:

(3) $$H_{min}=\frac{1}{a\cdot r}\cdot \sum_{i=1}^{a\cdot r}\;log_{2}\left(p_{imax}\right),$$

where $p_{imax}$ is the maximum probability of the *i*-th bit taking logic value ‘0’ or ‘1’ in the *k* responses [33]. If $H_{min}$ is 1, the *k* PUF responses are 100% independent and there are no correlations between the bits in different responses. This is the ideal situation to generate Helper Data that are quite independent and do not reveal information about the secret.

The minimum entropy of the impostor responses shown in Figure 4, corresponding to nominal operating conditions (25 °C), calculated as in Equation (3), is 91.92%. This means that the secrets are almost fully obfuscated because the responses are highly random.

**Figure 5:**
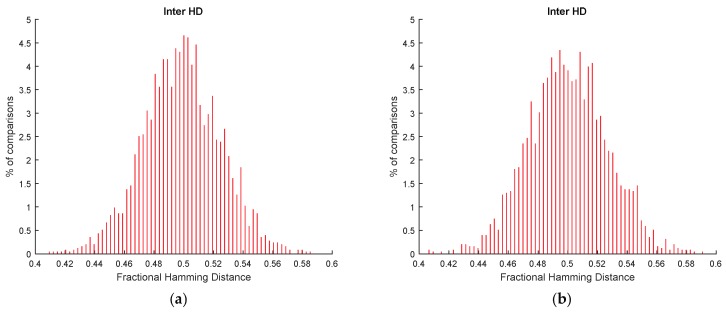
Inter Fractional Hamming Distance of Helper Data obtained: (**a**) with the same secret key for all the cameras; (**b**) with different key for each camera.

Considering PUF responses with ID cells generated at nominal operating conditions, we have studied the Helper Data generated by employing either the same secret key for all the cameras or a different secret key for each camera. In both cases, the Helper Data associated with each camera are different. Figure 5 illustrates the inter HD distributions of both Helper Data. An interesting result is that they are very similar (they are centered at 0.5 within a similar range). Therefore, if an attacker could extract the Helper Data of the cameras, he/she could not know if they are associated or not to the same secret key.

As described in Algorithm 4, the decoder of the ECC should cope with the noise of PUF responses to reconstruct the secret key without failures from the Helper Data. The bit flipping of a start-up value can be modeled essentially as a Bernoulli trial, which takes value ‘1’ (if the bit changes) with probability *p* and a value of ‘0’ (if the bit does not change) with probability 1 − *p*. If the *n* bits obtained from the start-up values of *n* cells are assumed to be independent, the probability of finding *t* flipping bits (or errors) in them is given by a binomial distribution. Hence, the probability that a PUF response of *n* bits contains more than *t* flipping bits is given by:

(4) $$P_{total}=1-\sum_{i=0}^{t}\left(\begin{array}{c} n\\ i \end{array}\right)\cdot p^{i}\cdot {\left(1-p\right)}^{n-i}.$$

The probability $P_{total}$ provides the failure probability in reconstructing a bit of the secret key when using an ECC with *n*-bit codewords and capacity to correct up to *t* errors, with *p* estimated by the average fractional HD of the genuine population [33,37]. For a given $P_{total}$, the number of errors (flipping bits) to be corrected and, hence, the complexity of the ECC, decreases as the value of the average fractional HD for the genuine population gets lower. This is why using only ID cells instead of all SRAM cells is advantageous.

The average fractional value of intra HD distribution obtained with ID cells (shown in Figure 4 above) is 0.0261. If this value is employed for *p* in Equation (4), an 8-bit repetition Error Correcting Code (with n=8 and t=3) gives a probability of failure in reconstructing a bit of the secret key *K* of 2.99 × 10^-5^ (according to the operation 1-binocdf (3, 8, 0.0261) in MATLAB 2017b from MathWorks®). Therefore, the probability to find some error in a 128-bit secret key is 0.0038 (1-binocdf (0, 128, 2.99 × 10^−5^)). For a 16-bit repetition Error Correcting Code, Equation (4) is evaluated by considering n=16 and t=7 since the probability of failure in reconstructing a bit of the secret key *K* would be equivalent to the probability that 8 bits of ${\bar{K}}_{coded}$ differ from $K_{coded}$ in eight bits or more. The resulting value for the probability is 2.30 × 10^−9^ (according to the operation 1-binocdf (7, 16, 0.0261)). Therefore, the probability to find some error in a 128-bit secret key is 2.94 × 10^−7^ (1-binocdf (0, 128, 2.30 × 10^−9^)). Hence, a 16-bit repetition Error Correcting Code is selected since an error rate of 10^−6^ is considered by many authors as a conservative value that fulfills the requirements of most of the typical security applications [37,47].

**Figure 6:**
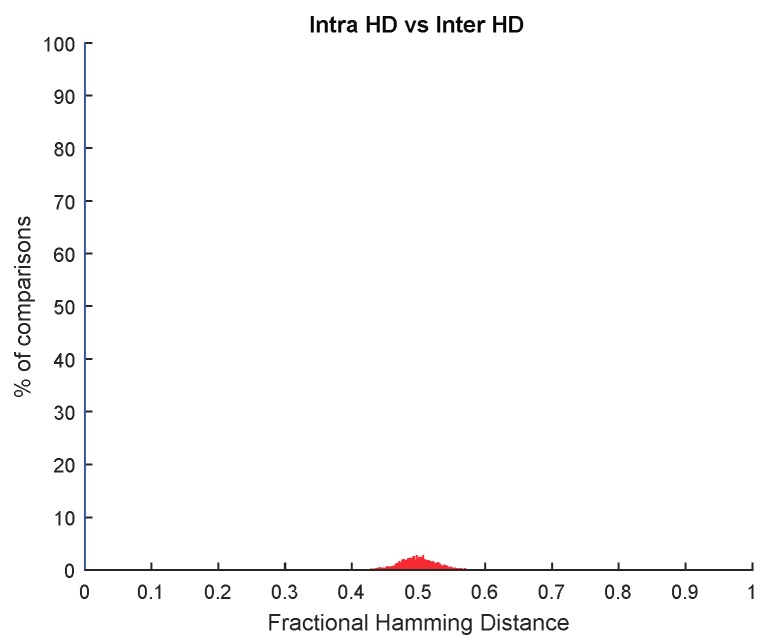
Fractional Hamming Distances between the correct secret key and the secret keys reconstructed by genuine cameras (on the left) and by impostor cameras (on the right).

In order to evaluate experimentally, the key reconstruction step with a 16-bit repetition ECC, the 100 measurements from the five BLE chips and the three temperatures were employed to reconstruct the secret key from the Helper Data associated with each device and calculated with the same secret key. The Hamming Distances calculated between the correct secret key and the reconstructed secret key when a genuine camera is employed (the secret key is reconstructed with the Helper Data and the PUF response of a genuine camera) are all zero, as shown on the left side of Figure 6. On the other side, the Hamming Distances calculated between the correct secret key and the reconstructed secret key when an impostor camera is employed (the secret key is reconstructed with the Helper Data of another camera and the PUF response of the impostor camera) are not zero but are centered at 0.5, as shown on the right side of Figure 6.

Table 1 shows a comparison with other proposals in the literature also aimed at securing cameras with hardware-based PUFs [24,25,26]. Our proposal is preferred whenever the hardware of the camera cannot be designed specifically to integrate the PUF into the image sensor [24,25] or into the controller of the virtual sensor node [26]. Our proposal can be applied to any camera on a mobile device by registering it and updating its firmware with the proposed trustworthy protocol described in Section 4 in order to execute the algorithms described in Section 3.

In all of the proposals shown in Table 1, the PUF uniqueness, evaluated by the average inter HD, shows a value close to the ideal value of 0.5. The PUF reliability, evaluated by the worst-case average intra HD, changes more. In [24], a high reliability can be obtained by increasing the threshold $P_{th}$. For that purpose, $P_{th}$ should be empirically determined and adjusted based on the characterization of the sensor fabrication process. In [25], reliability was calculated with simulation results considering variations of the operation conditions. In [26], reliability was measured with only nominal operation conditions. Reliability of our proposal (measured experimentally under variations of nominal operation conditions) can be further improved if the extraction of STB_mask considers measurements at all the range of temperatures, as described in [33]. Anyway, since the achieved reliability is good and can be managed by a simple repetition ECC, only measurements at nominal temperatures have been considered in Algorithm 1 in order to carry out the enrollment phase faster. The computational cost of applying a repetition ECC is much lower than applying BCH codes, as employed by other authors [25].

### 5.3. Evaluation of Nonces Extracted from SRAM PUFs Included in Cameras

The RND_masks for each BLE chip were created with the first 20 measurements at 25 °C, executing Algorithm 1. The RND cells were the cells taking ‘0’ ten times and ‘1’ the other ten. The minimum number of RND cells found in the BLE chips was 265 (0.47% of the 56,000 bits evaluated). This quantity is enough to generate the 128-bit nonces.

**Figure 7:**
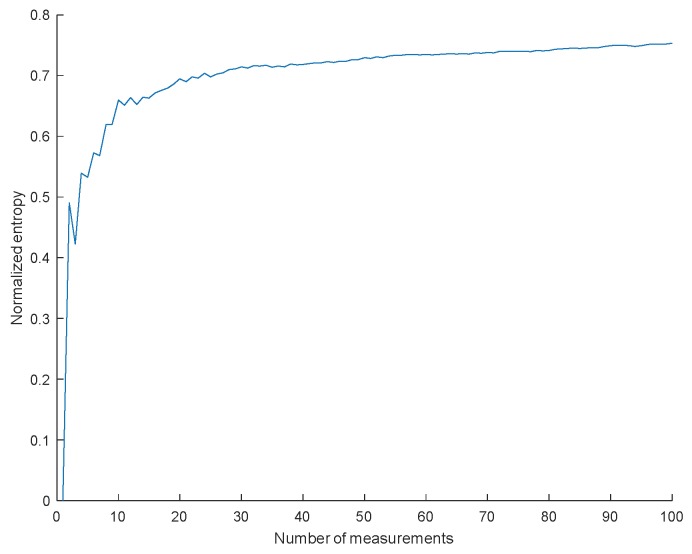
Convergence of the minimum entropy to its asymptotic value for responses of RND cells.

The sequences generated by the same RND cells at different start-ups (considering 10 BLE chips) were compared in pairs to calculate the average fractional Hamming distances. The result was 0.48, which is almost the ideal value of 0.5 for random sequences. The minimum entropy calculated as in Equation (3) with 100 measurements (from 1 to 100 start-ups) from the RND cells of one of the devices at nominal temperature converges to 75%, as shown in Figure 7.

The 100 sequences generated by the RND cells of one BLE chip after 100 power-up measurements at nominal temperature was evaluated by the NIST test suite for randomness [48]. The result of applying the frequency, forward and backward cumulative sums, and runs NIST tests to these sequences are shown in Table 2. The frequency test measures if the number of ones and zeros in the sequences are approximately the same as would be expected for a truly random sequence. This test is passed if the Hamming weight of the nonces tended to be 0.5, that is, the number of ones and zeros tended to be the same and there is no bias. The cumulative sums of the bits in the subsequences that can be formed from the complete sequence (considering ‘1’ and ‘−1′ values) should be zero, as in the complete sequence. In the forward mode, the subsequences are formed from the beginning to the end of the complete sequence, and, in backward mode, the subsequences are formed from the end to the beginning. The cumulative test measures if the subsequences of an unpredictable sequence are also unpredictable. The runs test measures if the oscillation among substrings of consecutive ones and consecutive zeros is too fast or too slow.Columns 1–10 in Table 2 correspond to the frequency of *p*-values (the unit interval is divided into ten discrete bins). Column 11 is the *p*-value that arises via the application of a chi-square test. Each NIST test is statistical and the *p*-values of a set of sequences are obtained. The proportion of sequences that pass a statistical test should fall inside the confidence interval (in this case, it is of 99%). Column 12 is the proportion of binary sequences that passed. The minimum pass rate for each statistical test is approximately 96 for a sample size of 100 binary sequences. Therefore, the bit strings provided by the RND cells of the BLE chips passed the basic NIST tests for randomness.

These measurements prove that BLE chip was able to generate nonces with 128 bits to be employed in the initialization vector and the key derivation functions.

### 5.4. Proof of Concept of a Trusted Camera

**Figure 8:**
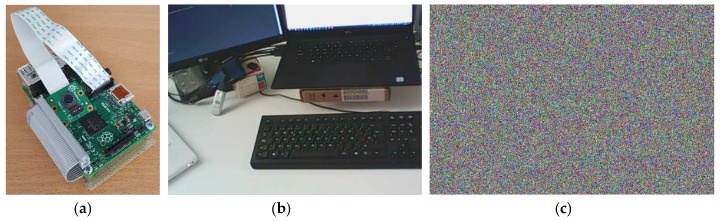
(**a**) prototype of a trusted camera; (**b**) original image acquired; (**c**) authenticated encrypted image.

A low-cost camera based on Raspberry Pi 2 model B was developed as a proof of concept. It employs an 8-megapixel sensor (Raspberry Pi Camera Module v2), which can provide video as well as still photographs. As BLE communication, it employs the CC2541 module connected to the GPIOs (General Purpose Input/Outputs) of the Raspberry. Figure 8a shows a photograph of the prototype. 

Picamera library for Python was used to work with the sensor module. The RPi.GPIO library allows working easily with the GPIO to power down and up the BLE chip and to extract the start-up values of the SRAM. A firmware implements the algorithms described in Section 3 to carry out the enrollment and normal operation phases. The PyCrypto library was used to manage the block cipher AES using a 128-bit secret key to encrypt and decrypt data as well as the CBC mode using a 128-bit initialization value.

As an example, an image taken by the camera and its corresponding ciphered result are illustrated in Figure 8b,c, respectively. The Tkinter library was employed to develop a Graphical User Interface to visualize the results.

## 6. Conclusions

The security of the control actions and firmware updates of a camera is increased by the proposal of a trusted camera and a communication protocol based on SRAM PUFs. The solution has been carefully designed to satisfy the constraints of low-cost cameras included in mobile devices by selecting lightweight and standard cryptographic algorithms and by avoiding additional hardware. We have analyzed SRAM PUFs extracted from commercial BLE chips which can be typically included in the communication module of a camera. Camera identifiers extracted from these BLE chips allow obfuscating sensitive 128-bit secret keys into non-sensitive Helper Data. Only the genuine trusted camera can recover the secret key from the Helper Data when it is required (thus, authentication is satisfied). In addition, 128-bit nonces are extracted from the SRAMs of the BLE chips. The secret keys and the IVs generated are employed for the standard authenticated encryption algorithm AES-CBC. Nonces are employed to derive session information by means of a Key Derivation Function based on AES. Confidentiality and integrity are satisfied by means of the authenticated encryption. Freshness is satisfied by means of the derivation of session information. The evaluation of the SRAM PUFs extracted from the BLE chips proves the properties of uniqueness, reliability, randomness and unpredictability. In spite of considering measurements from the BLE chips under temperature variations and a simple repetition Error Correcting Code, the secret keys are recovered perfectly. As a proof of concept, a Raspberry Pi-based mobile camera was developed, which contains the BLE chip as a communication unit that could be powered down and up to extract the SRAM PUF. In this work, we have detailed a security solution based on SRAM PUFs oriented to low-cost cameras. However, since SRAMs are included in many devices, the proposed solution can be applied to other low-cost devices. This will be considered as future lines of this work.

## Figures and Tables

**Table 1:** Comparison with other proposals using hardware-based PUFs.

Proposal	Worst-case Average Intra HD (%)	Average Inter HD (%)	Results	Specific Hardware
Frame-based image sensor [24]	12 ($P_{th}$ = 0) 0.2 ($P_{th}$ = 80)	49.37	Experimental, with variations of nominal conditions	Required
Event-based image sensor [25]	3.70	49.96	Simulated, with variations of nominal conditions	Required
Trusted visual sensor node [26]	1.40	~49.0	Experimental, with no variations of nominal conditions	Required
This work	2.61	49.67	Experimental, with variations of nominal conditions	Not required

**Table 2:** Basic NIST tests evaluated for the sequences provided by RND cells.

C1	C2	C3	C4	C5	C6	C7	C8	C9	C10	P-value	Proportion	Statistical Test
12	9	11	7	6	20	8	10	6	11	0.085587	96/100	Frequency
9	10	6	9	8	12	3	17	15	11	0.090936	96/100	CumulativeSums(fw)
10	7	8	6	7	22	4	9	12	15	0.003201	96/100	CumulativeSums(bw)
15	8	11	12	10	9	9	14	10	6	0.494392	98/100	Runs
